# Supplementary material for: Hybrid Models and Biological Model Reduction with PyDSTool
Source: PLoS Comput Biol. 2012 Aug 9;8(8):e1002628. doi: 10.1371/journal.pcbi.1002628 (PMC3415397; doi:10.1371/journal.pcbi.1002628)
Supplement: Text S4 — Complete source code for the PyDSTool package (version 0.88.120504). Includes API documentation and help files linking to web pages. This file is identical to the current public release on Sourceforge.net. (ZIP) [file pcbi.1002628.s004.zip › PyDSTool/html/PyDSTool.MProject.condition-class.html]

xml version="1.0" encoding="ascii"?


PyDSTool.MProject.condition


| Home | Trees | Indices | Help | PyDSTool |
| --- | --- | --- | --- | --- |

Package PyDSTool ::
Module MProject ::
Class condition

[hide private]

[frames] | no frames]

# Class condition

source code

```
object --+
         |
        condition
```

---

Model context condition, made up of a boolean composition of wanted
and unwanted features. This is specified by a dictionary of feature
objects mapping to True (wanted feature) or False (unwanted feature).


|  |  |
| --- | --- |
| Instance Methods | [hide private] |

|  |  |
| --- | --- |
| \_\_init\_\_(self, feature\_composition\_dict)  x.\_\_init\_\_(...) initializes x; see x.\_\_class\_\_.\_\_doc\_\_ for signature | source code |

|  |  |
| --- | --- |
| \_\_eq\_\_(self, other) | source code |

|  |  |
| --- | --- |
| \_\_ne\_\_(self, other) | source code |

|  |  |
| --- | --- |
| keys(self) | source code |

|  |  |
| --- | --- |
| values(self) | source code |

|  |  |
| --- | --- |
| items(self) | source code |

|  |  |
| --- | --- |
| \_\_getitem\_\_(self, name) | source code |

|  |  |
| --- | --- |
| \_\_contains\_\_(self, name) | source code |

|  |  |
| --- | --- |
| set\_ref\_traj(self, ref\_traj)  Set reference trajectory for the features (if used, otherwise will be ignored or overridden in feature \_local\_init methods). | source code |

|  |  |
| --- | --- |
| evaluate(self, target)  Apply conditions to trajectory segments and returns True if all are satisfied. | source code |

|  |  |
| --- | --- |
| \_\_call\_\_(self, target)  Apply conditions to trajectory segments and returns True if all are satisfied. | source code |

|  |  |
| --- | --- |
| \_\_str\_\_(self)  str(x) | source code |

|  |  |
| --- | --- |
| \_\_repr\_\_(self)  str(x) | source code |

|  |  |
| --- | --- |
| \_find\_idx(self) | source code |

|  |  |
| --- | --- |
| \_residual\_info(self)  Update metric information used for residual / objective function, from all sub-features. | source code |

|  |  |
| --- | --- |
| collate\_results(self, result\_name, merge\_lists=False, feature\_names=None) | source code |

**Inherited from `object`**:
`__delattr__`,
`__getattribute__`,
`__hash__`,
`__new__`,
`__reduce__`,
`__reduce_ex__`,
`__setattr__`


|  |  |
| --- | --- |
| Properties | [hide private] |

**Inherited from `object`**:
`__class__`


|  |  |
| --- | --- |
| Method Details | [hide private] |

|  |  |
| --- | --- |
| \_\_init\_\_(self, feature\_composition\_dict)  *(Constructor)* | source code |

x.\_\_init\_\_(...) initializes x; see x.\_\_class\_\_.\_\_doc\_\_ for
signature

Overrides: object.\_\_init\_\_: *(inherited documentation)*

|  |  |
| --- | --- |
| \_\_str\_\_(self)  *(Informal representation operator)* | source code |

str(x)

Overrides: object.\_\_str\_\_: *(inherited documentation)*

|  |  |
| --- | --- |
| \_\_repr\_\_(self)  *(Representation operator)* | source code |

str(x)

Overrides: object.\_\_repr\_\_: *(inherited documentation)*

  


| Home | Trees | Indices | Help | PyDSTool |
| --- | --- | --- | --- | --- |

|  |  |
| --- | --- |
| Generated by Epydoc 3.0.1 on Fri May 4 15:24:06 2012 | http://epydoc.sourceforge.net |
